# Migration and Safety Assessment of 20 Antioxidants in 39 Disposable Biodegradable Tableware Products

**DOI:** 10.3390/foods15050964

**Published:** 2026-03-09

**Authors:** Liqian Wang, Yuting Chen, Xiaomeng Gao, Wenjun Zhou, Guowei Ma, Jingwei Zhang, Di Feng

**Affiliations:** Key Laboratory of Geriatric Nutrition and Health, Beijing Technology and Business University, Ministry of Education, Beijing 100048, China; 17860395785@163.com (L.W.); chenyuting20231005@163.com (Y.C.); spgaoxiong@163.com (X.G.); zhouwenjun020603@163.com (W.Z.); maguoweisxl@163.com (G.M.); meowcree2022@163.com (J.Z.)

**Keywords:** disposable biodegradable tableware, UPLC-MS/MS, antioxidants, exposure assessment, migration

## Abstract

(1) Background: The safety of antioxidants (AOs) in disposable biodegradable tableware products remains insufficiently understood. (2) Methods: The migration of 20 AOs from 39 disposable biodegradable tableware under multiple usage conditions was investigated by Ultra Performance Liquid Chromatography-Tandem Mass Spectrometry. Their potential exposure risks were evaluated using three risk assessment frameworks (EU, FDA, and Monte Carlo simulation). (3) Results: Ten AOs were detected in 95% ethanol, with Irganox 1010 showing the highest migration (0.29 ± 0.62 mg/kg). Starch-based products exhibited a greater variety and higher migration of AOs compared to PLA-based and fiber-based products. Food simulant type, temperature, and time exerted a more significant effect on AO migration than microwave and ultraviolet treatments. An analysis method for six typical AOs in soybean oil using freezing degreasing was established, which demonstrated good recoveries (77.6–110.3%) and relative standard deviations (1.7–14.7%). Four AOs were detected in soybean oil, with Irganox 1010 showing the highest migration (603.7 × 10^−3^ mg/kg). Utilizing high-percentile conservative exposure scenarios derived from Monte Carlo simulation, Irganox 1010 may pose a health risk to humans under high-dose exposure in soybean oil. (4) Conclusions: This study provides a basis for the safety evaluation of AOs in disposable biodegradable tableware.

## 1. Introduction

Food contact materials (FCMs) are vital for the storage and transportation of food, making their safety a key component of overall food safety [1]. In response to the global movement to ban plastic products, biodegradable plastics have been widely promoted as alternatives to conventional petroleum-based plastics. However, processing additives such as antioxidants and plasticizers are commonly used to enhance their performance. These additives may migrate from biodegradable FCMs into food, potentially posing health risks to humans [2,3,4].

Synthetic antioxidants (AOs) are widely used to prevent the oxidative degradation of polymer materials, thereby extending their durability. AOs typically include primary and secondary antioxidants, which are used in combination to enhance the overall antioxidant effect. Primary antioxidants, such as phenolic and amine antioxidants, function by interrupting free radical chain reactions that lead to oxidation [5]. Among these, Irganox 1010 and Irganox 1076 are the most commonly used synthetic phenolic antioxidants (SPAs). Secondary antioxidants, such as the prevalent phosphite ester antioxidant Irgafos 168, reduce oxidation by decomposing peroxides that generate radicals [6,7]. It is important to note that AOs do not form covalent bonds with polymers, which may result in the migration of residual AOs and their degradation products into food during usage, thereby posing potential health risks to humans [8,9]. Research has demonstrated that certain AOs exhibit toxicological effects in animal studies, including reproductive and developmental toxicity, DNA damage, endocrine-disrupting effects, and tumor promotion [10,11,12,13]. Furthermore, various AOs, including Irganox 1010, Irganox 1076, BHT, 2,4-DTBP, and Antioxidant 2246, have been detected in multiple human biological samples, such as urine, breast milk, fingernails, serum, and umbilical cord serum, indicating widespread human exposure to AOs [14,15,16,17,18].

As emerging contaminants, AOs in FCMs have been found to migrate into food and food simulants, raising concerns about exposure risks. Generally, human exposure to AOs is assessed by estimating the daily intake (EDI) based on migration concentrations. Common deterministic assessment methods, including those used by the European Union (EU) and the U.S. Food and Drug Administration (FDA), rely on fixed parameters to generate single-point EDI estimates for routine screening. In contrast, probabilistic assessments employ Monte Carlo simulations with variable parameters to quantify exposure variability and uncertainty; these are typically applied when deterministic results indicate potential concerns [7]. Irganox 1010, Irganox 1076, and 2,4-DTBP have been detected in infant milk powder and edible oils [19]. Ji et al. reported that the concentration range of 23 AOs in food was higher than that found in both the inner and outer packaging. Their estimated daily intake (EDI) for adolescents and adults was calculated to be 2.55 × 10^−2^ and 1.24 × 10^−2^ mg/kg bw/day, respectively [20]. Liu et al. identified Irgafos 168, Irganox 1076, Irganox 1010, and Antioxidant DLTP migrating from seven traditional plastics into migration simulants, with concentrations ranging from 1.2 to 388.1 ng/mL. Risk assessments using Monte Carlo simulations indicated that the safety risk of AOs in polypropylene FCMs is relatively high [7]. Two studies on the long-term migration of AOs from traditional plastic FCMs into edible oils also detected Irganox 1010, with the highest migration level reaching 13.35 mg/kg [21,22]. Additionally, some studies have shown a higher potential for additive leaching from biodegradable films compared to traditional plastic films [23]. However, current research on AO migration in FCMs predominantly focuses on traditional plastic materials. Data on the migration behavior and safety assessment of AOs in biodegradable FCMs across multiple food simulants, especially in real edible oil systems, remain limited.

Studies have shown that AOs may pose a higher risk of exposure when disposable tableware comes into contact with high-temperature and fatty foods [6,9]. Sales of disposable biodegradable tableware are rapidly increasing due to growing environmental awareness and the rise in takeaway food consumption. Because of their cost-effectiveness and cooking performance, most restaurants in China use soybean oil and rapeseed oil for cooking. Therefore, it is urgent to investigate the migration and exposure risks of AOs when disposable biodegradable tableware comes into contact with edible oils. The migration of AOs from FCMs has been widely detected using ultra-performance liquid chromatography-tandem mass spectrometry (UPLC-MS/MS). The complexity of the oil matrix necessitates the implementation of an efficient sample pretreatment method for purification and degreasing. Current pretreatment techniques for the analysis of AOs in oil-based foods primarily include deep eutectic solvent extraction [24], QuEChERS [25], solid-phase extraction [26], and the frozen degreasing method [9]. Among these, the frozen degreasing method, which removes fat by applying low temperatures, is a simpler, more cost-effective, and reliable sample preprocessing technique. For instance, Du et al. employed this method coupled with UPLC-MS/MS to analyze 18 AOs in 10 edible oils, achieving recoveries ranging from 68% to 118% and precision between 2% and 18% [9].

This study analyzed the migration of 20 AOs from 39 disposable biodegradable tableware into a fatty food simulant (95% ethanol) using UPLC-MS/MS, and the effects of various usage conditions on AO migration were systematically investigated. Soybean oil was employed to conduct accelerated migration tests, and an optimized frozen degreasing method was employed for sample pretreatment. The migration levels of AOs in soybean oil from representative samples were quantified. Finally, exposure risks associated with AOs were assessed through both deterministic assessment (EU and FDA method) and probabilistic assessment (Monte Carlo simulation). In summary, this study provides a scientific foundation for evaluating the migration and safety risks of 20 AOs in disposable biodegradable tableware.

## 2. Materials and Methods

### 2.1. Sample Collection

A total of 39 disposable biodegradable tableware products were purchased from Chinese markets. These products include the three most commonly utilized biodegradable materials in the market. They were obtained from stores specializing in disposable tableware for over five years, with purchase records exceeding 5000 transactions. Based on product descriptions, the items were classified into three categories: PLA (Polylactic Acid)-based products (*n* = 10), starch-based products (*n* = 15), and fiber-based products (*n* = 14). The sample set comprised meal boxes (*n* = 10), dinner plates (*n* = 10), cups (*n* = 6), forks (*n* = 4), straws (*n* = 3), bowls (*n* = 3), a tea bag (*n* = 1), a plastic wrap (*n* = 1), and a bag (*n* = 1). Detailed information regarding the tableware is presented in Table S1. Prior to analysis, all samples were stored at room temperature and had not been previously exposed to food.

### 2.2. Reagents

Twenty AOs were analyzed, including 17 AOs permitted for use in FCMs in China [27], as well as two degradation products and one parent compound commonly found in FCMs. Twenty AO standards were purchased from TCI (Shanghai) Development Co., Ltd. (Shanghai, China) with purities > 95%. Detailed information, including the names, abbreviations, CAS registry numbers, and permitted uses of these standards, is presented in Table S2. These AOs have been reported as detectable in the literature and are commercially available. LC-MS grade methanol (MeOH), acetonitrile (ACN), ammonium acetate, and LC grade dichloromethane (DCM) for UPLC-MS/MS analysis were obtained from Fisher Scientific (Waltham, MA, USA). Water was prepared using a Milli-Q ultrapure water system (Millipore, Woburn, MA, USA). Acetic acid and ethanol (analytical pure) were purchased from Beijing Chemical Works (Beijing, China). Soybean oil was purchased from local markets in Beijing, China.

### 2.3. Migration Test

#### 2.3.1. Migration Test in Food Simulants

Migration tests were performed in accordance with EU Regulation No. 10/2011 [28]. Given the diverse range of food types that disposable biodegradable tableware may encounter, the following simulants were selected: 3% acetic acid to represent acidic foods (e.g., apple juice, lemon juice); 10% ethanol to represent aqueous and alcoholic foods (e.g., beer, wine); 50% ethanol to represent dairy products (e.g., milk and dairy beverages); and 95% ethanol to simulate fatty foods (e.g., oil-based soups, soybean oil).

Each sample (6 cm^2^) was immersed in 10 mL of food simulant and subjected to migration testing at 70 °C for 2 h as an accelerated migration test. Triplicate experiments were conducted alongside simultaneous blank controls. Upon completion of the migration tests, the migration solutions were cooled to room temperature. Then, 1 mL of each migration solution was filtered through a 0.22 μm polytetrafluoroethylene (PTFE) membrane and prepared for UPLC-MS/MS analysis.

The effects of various conditions on the migration of AOs were evaluated based on the actual usage of the products. These conditions examined included: (1) food simulants (3% acetic acid, 10%, 50%, and 95% ethanol); (2) migration temperatures (4, 20, 30, 50, and 70 °C); migration time (0.5, 1, 2, 6, and 24 h); (3) microwave exposure: 700 W output power with exposure time (0, 1, 2, 3, 4, and 5 min); (4) output powers (0, 140, 280, 420, 560, and 700 W) with 3 min exposure; and (5) ultraviolet irradiation durations at 254 nm (0, 15, 30, 45, and 60 min). For conditions (3)–(5), following the specified treatments, the samples were subjected to accelerated migration tests at 70 °C for 2 h in 95% ethanol.

#### 2.3.2. Migration Test in Soybean Oil

Owing to its cost-effectiveness and favorable cooking properties, soybean oil is predominantly utilized in Chinese restaurants. To simulate the interaction between tableware and fatty Chinese cuisine, soybean oil was used for simulated migration experiments. Each sample, measuring 6 cm^2^, was immersed in 10 mL of soybean oil and subjected to migration at 70 °C for 2 h. After migration, 0.25 ± 0.01 g of soybean oil and 5 mL of ACN were placed into a 10 mL glass centrifuge tube sealed with a stopper. The mixture was vortexed for 3 min and ultrasonicated for 15 min, then centrifuged at 2860× *g* for 5 min. The upper ACN layer was carefully collected, and the extraction was repeated with an additional 5 mL of ACN. The combined ACN extracts were evaporated to near dryness, reconstituted in 2 mL of ACN and stored at −80 °C for 60 min. The supernatant was filtered through a 0.22 μm PTFE membrane and prepared for analysis by UPLC-MS/MS.

### 2.4. Instrumental Analysis

A Waters ACQUITY™ UPLC system, coupled with a Waters Xevo TQ-S Micro MS system (Waters, Milford, MA, USA), and a Waters ACQUITY BEH C_18_ column (2.1 × 100 mm, 1.7 μm), was employed for the analysis. The column temperature was maintained at 40 °C. The mobile phase consisted of 1 mM ammonium acetate solution (A) and MeOH (B). The gradient elution program was as follows: an initial composition of 20% B was held for 0.2 min, followed by an increase to 100% B over 2.5 min, which was then maintained for 4.5 min, concluding with equilibration at 20% B for 2 min. The injection volume was 5 μL, and the flow rate was 0.3 mL/min. Electrospray ionization (ESI) was performed in multiple reaction monitoring (MRM) mode, simultaneously scanning both positive (ESI+) and negative (ESI-) ion modes, with the capillary voltage set at 4.5 kV for both modes. Ion source temperature was 150 °C; desolvation temperature was 500 °C; desolvation gas flow rate was 1000 L/h. Data acquisition and processing were performed using the Waters MassLynx 4.1 software package. The mass spectrometric parameters for the 20 AOs are shown in Table 1.

### 2.5. Methodological Validation

#### 2.5.1. Preparation of Standard Curves

An AO standard weighing 0.0050 ± 0.0001 g and methanol were placed in a 50 mL volumetric flask to prepare a standard stock solution with a concentration of 100 μg/mL. For migration test in solvent-based food simulants, this stock solution was subsequently diluted with 3% (*v*/*v*) acetic acid, 10% ethanol, 50% ethanol, 95% ethanol, respectively, to prepare 20 AOs matrix-matched standard solutions with concentrations ranging from 0.5 ng/mL to 2000 ng/mL. For migration test in soybean oil, 200 μL standard stock solution was added to 20 g blank soybean oil to prepare an oil sample containing Irganox 1310, Antioxidant JX-35, Irganox 1076, Irganox 1010, 2,4-DTBP, and Irgafos 168 at a concentration of 1000 μg/kg. Then they were gradually diluted with soybean oil to obtain AOs contents ranging from 10 μg/kg to 1000 μg/kg. After sample pretreatment, they were analyzed by UPLC-MS/MS to generate matrix-matched calibration curve.

#### 2.5.2. Methodological Evaluation

The calibration curves, linear ranges, correlation coefficients (R^2^), limits of detection (LODs, signal-to-noise ratio of 3), limits of quantitation (LOQs, signal-to-noise ratio of 10), and relative standard deviations (RSDs, n = 6) for AOs were systematically evaluated.

For the sample preparation method of AOs in soybean oil, both solvent standard solutions and matrix-matched standard solutions, prepared at identical concentrations, were analyzed using UPLC-MS/MS to calculate the matrix effect (ME). The ME was calculated according to the following formula: (Slope of the matrix-matched calibration curve/Slope of the solvent calibration curve-1) × 100%. ME > 0 indicates matrix enhancement, while ME < 0 indicates matrix suppression. Specifically, |ME| < 20% indicates a weak matrix effect, 20% < |ME| < 50% indicates a moderate matrix effect, and |ME| > 50% indicates a strong matrix effect [29]. Additionally, spiked recovery experiments were performed at three concentration levels (0.02, 0.2, and 0.5 mg/kg), each with six replicates.

### 2.6. Exposure Assessment

#### 2.6.1. EU and FDA Methods

EDI value is utilized to assess the safety of pollutant exposure levels by comparing it with the No Observed Adverse Effect Level (NOAEL), thereby determining whether the exposure remains within a safe threshold. The NOAEL represents the quantitative hazard value obtained from the EPA Comp Tox Chemicals Dashboard database [30]. The Margin of Exposure (MOE) is applied for risk description. For AOs that have a defined health threshold (i.e., those lacking genotoxicity or carcinogenicity), MOE ≥ 100 is generally regarded as indicative of no significant health risk [31]. The MOE is calculated according to Equation (1).

(1) $$\begin{array}{c} MOE=\frac{\mathrm{NOAEL}}{\mathrm{EDI}} \end{array}$$

The methods for calculating the EDI differ across countries, primarily including the assessment approaches employed by the EU and FDA, as well as probabilistic models utilizing Monte Carlo simulations. In practical applications, it is generally assumed that the volume (L) of the food simulant is equivalent to the weight (kg) of the food, and that the migration amount corresponds to the dietary exposure concentration [32]. Specifically, the calculation methods for the EDI of pollutants in FCMs vary between the EU and FDA. The EU approach assumes that an individual weighing 60 kg consumes 1 kg of food daily, which is packaged in materials with a contact surface area of 6 dm^2^ [33]. The EDI is calculated according to Equation (2).

(2) $$\begin{array}{c} EDI=\frac{M\;\times \;\mathrm{fc}}{\mathrm{BW}} \end{array}$$

where M is the migration amount of AOs (mg/kg); fc (food consumption) = 1 kg food/per/d; BW (body weight) = 60.95 kg [34].

The FDA method assumes a daily food consumption of 3 kg per person. Additionally, it considers the Consumption Factor (CF) and the food type distribution factor (f_T_) [32]. The EDI is calculated using Equations (3) and (4).

(3) $$\begin{array}{c} \text{<}M\text{>}=f_{\mathrm{aqueous}}M_{10\%\;\mathrm{ethanol}}+f_{\mathrm{alcohol}}M_{50\%\;\mathrm{ethanol}}+{\;f}_{\mathrm{fatty}}M_{95\%\;\mathrm{ethanol}}+f_{\mathrm{acetic}}M_{3\%\;\mathrm{acetic}\;\mathrm{acid}} \end{array}$$

(4) $$\begin{array}{c} EDI=\frac{\text{<}M\text{>}\;\times \;\mathrm{fc}\;\times \;\mathrm{CF}}{\mathrm{BW}} \end{array}$$

where M represents the migration amount (mg/kg); fc = 3 kg food/per/d; and f_T_ includes aqueous, acidic, alcoholic, and fatty foods. CF and f_T_ are presented in Table S3.

#### 2.6.2. Monte Carlo Simulation

Based on the Monte Carlo simulation method, the Oracle Crystal Ball risk analysis software package (version 11.1.2.4) was utilized to predict the exposure of EDI and the Hazard Quotient (HQ) [7]. Exposure parameters and models (Table S4) were applied, with 10,000 iterations conducted for each simulation. The probability assessment model is presented in Equation (5).

(5) $$\begin{array}{c} EDI=\frac{C\;\times \;\mathrm{fc}\;\times \;F_{\mathrm{abs}}\;\times \;\mathrm{EF}\;\times \;\mathrm{ED}}{\mathrm{LT}\;\times \;\mathrm{BW}} \end{array}$$

where C is the dietary concentration (mg/kg), equivalent to the migration amount; F_abs_ is the absorption factor; EF: exposure frequency (d); ED: exposure duration (years); LT (lifetime) = ED × 365 (d/year). Detailed parameters are provided in Table S4. F_abs_ is calculated using Equation (6) [35].

(6) $$\begin{array}{c} F_{\mathrm{abs}}=\frac{1}{1.11\;\times \;10^{-8}\;\times {\;K}_{\mathrm{ow}}+\left(0.41/K_{\mathrm{ow}}\right)+1.01} \end{array}$$

The octanol/water partition coefficient (K_ow_) was obtained from the EPA CompTox Chemicals Dashboard database [30], with specific values detailed in Table S5. HQ is used to describe risk. HQ < 1 suggests that the AOs pose negligible health risks to the exposed population. Conversely, HQ ≥ 1 indicates that exposure levels of the component may lead to potential human health risks [7]. The calculation of HQ is conducted using Equation (7).

(7) $$\begin{array}{c} HQ=\frac{\mathrm{EDI}}{\mathrm{RfD}} \end{array}$$

The Reference Dose (RfD) serves as a health reference dose for exposure assessment and is derived from the NOAEL recommended by the European Food Safety Authority (EFSA) or obtained through the Threshold of Toxicological Concern (TTC) approach with the Toxtree software (version 3.1.0) application. Specific values are provided in Table S5.

### 2.7. Statistical Analysis

The IBM SPSS software package (version 20; IBM Corp., Armonk, NY, USA) was used to calculate the mean, standard deviation, and to determine the probability distribution of the dataset. Pearson correlation analysis was performed using the Correlation Plot plugin in Origin (version 2019; OriginLab Corp., Northampton, MA, USA) .

## 3. Results and Discussion

### 3.1. Simulated Migration Results of AOs from 39 Products into 95% Ethanol

**Figure 1:**
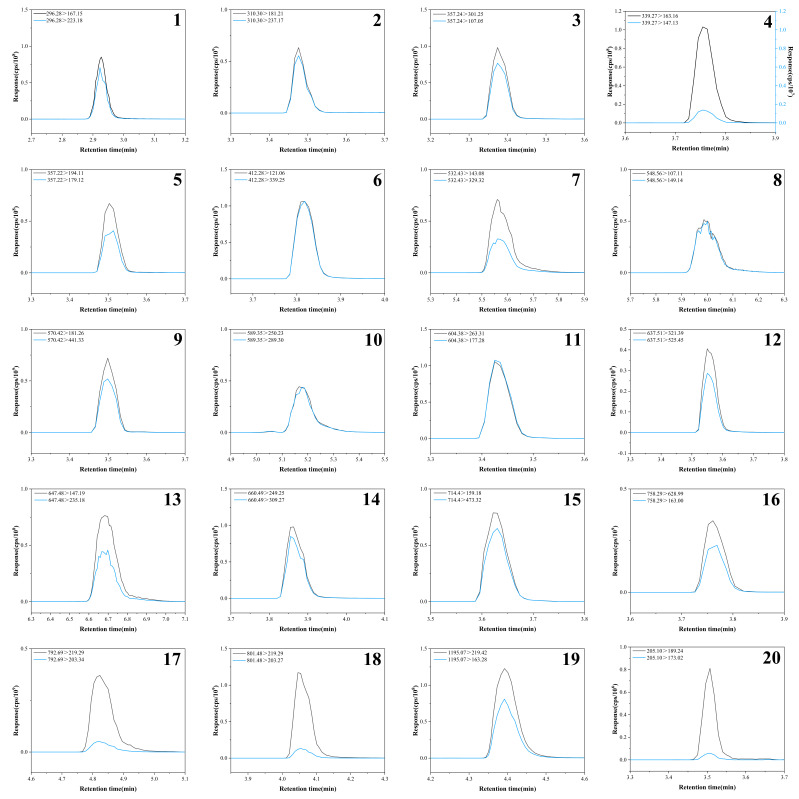
Extracted ion chromatograms of 20 AOs standards. (**1**) Irganox 1310; (**2**) Antioxidant JX-35; (**3**) Irganox 1222; (**4**) Antioxidant 2246; (**5**) Antioxidant 300; (**6**) Irganox 3052; (**7**) Antioxidant DLTP; (**8**) Irganox 1076; (**9**) Irganox 1024; (**10**) Irganox 565; (**11**) Irganox 245; (**12**) Irganox 1098; (**13**) Irgafos 168; (**14**) Irganox 1035; (**15**) Irganox 697; (**16**) Irganox 80; (**17**) Irganox 330; (**18**) Irganox 3114; (**19**) Irganox 1010; (**20**) 2,4-DTBP.

Matrix-matched calibration curves were employed for four solvent-based food simulants to compensate for potential matrix-induced ion suppression or enhancement, thereby ensuring accurate quantification. Table S6 presents the calibration curves, linear ranges, R^2^, LODs, LOQs, and RSDs for 20 AOs across the four food simulants. The findings demonstrate that all 20 AOs exhibit strong linearity (R^2^ > 0.99) within the tested simulants. The LODs range from 0.0001 × 10^−3^ to 3.77 × 10^−3^ mg/kg, while the LOQs range from 0.0002 × 10^−3^ to 12.57 × 10^−3^ mg/kg. The RSDs range from 1.2% to 9.2%. The extracted ion chromatogram of the mixed standards for the 20 AOs is illustrated in Figure 1.

The most extreme usage conditions for disposable tableware involve exposure to high temperatures and high-fat food. Research has further demonstrated that the migration levels of AOs in 95% ethanol more closely resemble those observed in olive oil compared to other fatty food simulants, such as isooctane and n-heptane. Therefore, 95% ethanol is considered a more appropriate simulant for exposure assessment [28]. Consequently, a simulated migration experiment was performed utilizing 95% ethanol at 70 °C for 2 h, and the migration levels of 20 AOs from 39 products were analyzed (Table 2). The results indicated the presence of AOs in 20 samples, yielding a detection rate (DR) of 51%. Notably, 16 of these samples contained two or more antioxidants. The DR of AOs across the three product categories were ranked as follows: starch-based (100%) > PLA-based (50%) > fiber-based (0%). Furthermore, each starch-based product contained two or more types of AOs, with two samples exhibiting the simultaneous detection of up to seven AOs. Based on detection rates and AOs variety, the potential safety risks associated with AOs in the three product categories are ranked as follows: starch-based > PLA-based > fiber-based.

A total of ten AOs were identified, including seven hindered phenols: Irganox 1310, Antioxidant JX-35, Irganox 1076, Irganox 245, Irganox 3114, Irganox 1010, and 2,4-DTBP; one phosphite, Irgafos 168; one thioester, Antioxidant DLTP; and one hindered amine, Irganox 330. Among these, Irganox 1010, Irgafos 168, and Irganox 1076 exhibited elevated migration levels, with average migration amounts of 0.29 ± 0.62, 0.26 ± 0.73, and 0.063 ± 0.12 mg/kg, respectively. The maximum migration amounts recorded for these substances were 2.77, 4.23, and 0.44 mg/kg, respectively. These findings indicate that Irganox 1010 and Irganox 1076 are the predominant AOs commonly utilized in biodegradable FCMs, frequently in combination with the secondary antioxidant Irgafos 168. The specific migration limits (SML) for Irganox 1076, Antioxidant DLTP, Irganox 3114, and Irganox 245 are 6, 5, 5, and 9 mg/kg, respectively. For AOs lacking defined SMLs, a default limit of 60 mg/kg is generally applied [27,28]. Additionally, the recommended SML for Irgafos 168 is 10 mg/kg [36]. The results show that the migration levels of the detected AOs do not exceed their respective SMLs.

**Figure 2:**
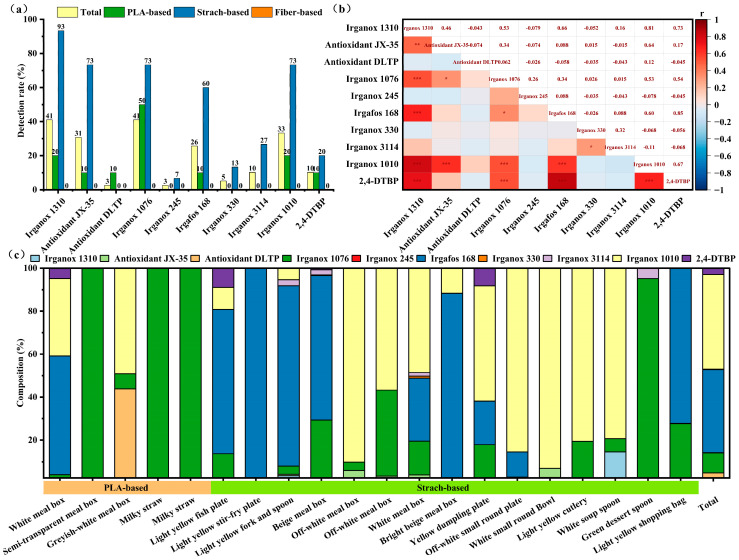
Analysis results of AOs migration from 39 disposable biodegradable tableware products to 95% ethanol. (**a**) Detected rate of AOs; (**b**) Pearson correlation analysis of AOs migration amount; (**c**) composition characteristics of AOs in 20 samples. Significance levels are represented with asterisk as following: * represents *p* < 0.05; ** represents *p* < 0.01; *** represents *p* < 0.001.

The DRs of the ten AOs are presented in Figure 2a. Irganox 1310 and Irganox 1076 exhibited the highest total DRs, each at 41%, followed by Irganox 1010 at 33%, Antioxidant JX-35 at 31%, and Irgafos 168 at 26%. In PLA-based products, the AOs with higher DRs included Irganox 1076 (50%), Irganox 1310 (20%), and Irganox 1010 (20%). In starch-based products, significant detection rates were observed for Irganox 1310 (93%), Antioxidant JX-35 (73%), Irganox 1076 (73%), and Irganox 1010 (73%). Pearson correlation analysis of the migration amounts of the detected AOs (Figure 2b) revealed a strong positive correlation (r = 0.60–0.85, *p* < 0.05) among the migration amounts of Irganox 1310, Antioxidant JX-35, Irgafos 168, Irganox 1010, and 2,4-DTBP, suggesting that these AOs may exhibit similar migration behaviors. Among these, Irganox 1010 and Irgafos 168 are commonly used primary and secondary antioxidants, respectively, and are typically combined to achieve a synergistic effect. Additionally, 2,4-DTBP and Irganox 1310 are degradation products of Irgafos 168 and Irganox 1010 [37,38], respectively. Antioxidant JX-35 is a residue of the parent compound of Irganox 1010. Furthermore, analysis of the compositional characteristics of AO migration amounts across 20 samples (Figure 2c) indicates that starch-based products exhibited the greatest diversity of AOs. Irganox 1010 contributed the largest proportion (44%) of total migration, followed by Irgafos 168 (39%) and Irganox 1076 (9%). Collectively, these three AOs accounted for 92% of the total migration observed. The higher migration levels and greater variety of AOs in starch-based products may be attributed to their unique structural and performance characteristics: the molecular chains of starch contain numerous hydroxyl groups, rendering them more susceptible to oxidation reactions. Consequently, compared to PLA and cellulose, starch-based products may require the addition of more AOs [39]. Additionally, starch-based materials tend to form porous structures during processing; the higher the starch content, the greater the porosity of the materials, which provides more channels for AO migration [40]. It is noteworthy that starch-based products typically contain blended polymers or coating components, which significantly impact the migration behavior of AOs. Therefore, the influence of the material matrix on AO migration warrants further investigation.

A migration study employing 95% ethanol was conducted on 257 samples involving seven types of traditional plastics—namely polypropylene, polyethylene, polyvinylidene chloride, polyethylene terephthalate, polyvinyl chloride, polystyrene, and polycarbonate —collected in China. The results revealed that Irgafos 168 exhibited the highest migration levels, ranging from 2.3 to 388.1 ng/mL, with a DR of 56%. Additionally, Irganox 1010, Irganox 1076, and Antioxidant DLTP were detected, with migration levels varying between 1.2 and 109.6 ng/mL [7]. In contrast, the biodegradable FCMs examined in this study released a greater diversity of AOs, totaling ten distinct types, and demonstrated higher migration levels, ranging from 6 × 10^−4^ to 4.23 mg/kg. Migration analysis conducted using 95% ethanol on 17 AOs across 118 samples representing five types of traditional plastics—namely polypropylene, polyethylene, polyethylene-laminated paper, polyethylene terephthalate, and laminated aluminum foil paper—obtained in China, indicated that 2,4-DTBP was the predominant SPA, with a maximum migration concentration of 44.39 ng/mL, followed by BHT-quinol (1.43 ng/mL), BHT-Q (1.37 ng/mL), and BHT (0.73 ng/mL) [4]. The findings of this study indicate that the migration levels of high-molecular-weight AOs were significantly higher than those of low-molecular-weight AOs reported in previous research. Therefore, enhanced scrutiny is necessary to ensure the safety of high-molecular-weight AO migration in biodegradable FCMs. In follow-up studies, in addition to exposure levels, toxicological characteristics and bioavailability should be comprehensively considered to evaluate the safety of AOs that have received significant attention, including their degradation and transformation products.

### 3.2. Influence of Different Usage Conditions on AOs Migration

**Figure 3:**
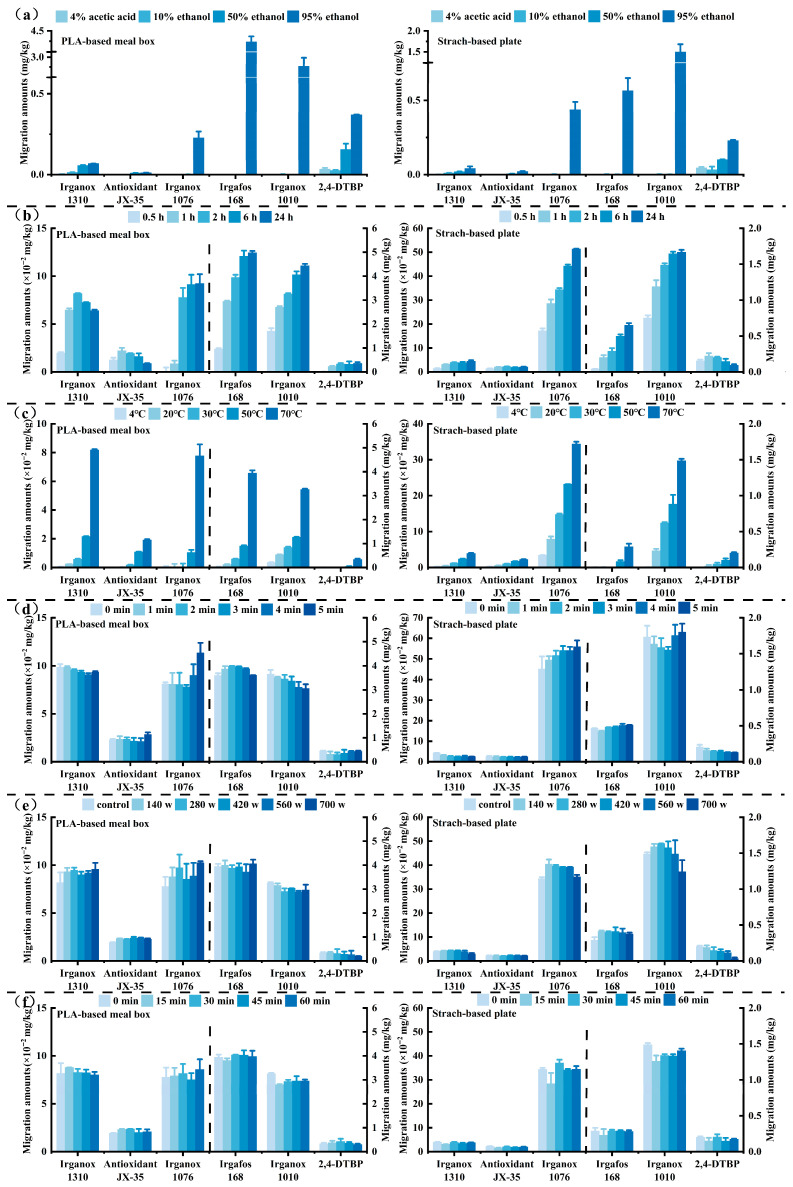
Impact of different usage conditions on the migration amounts of AOs. (**a**) Food simulants; (**b**) migration time; (**c**) migration temperature; (**d**) microwave time; (**e**) microwave power; (**f**) ultraviolet irradiation time (λ = 254 nm).

Given the greater variety and higher migration levels of AOs in 95% ethanol, a PLA-based meal box and a starch-based plate were selected as representative samples for this study. The study investigated the influence of food simulants, migration time and temperature, as well as microwave and ultraviolet treatments on the migration of AOs (Figure 3).

The migration levels of six AOs detected in two products across four food simulants were ranked in descending order as follows: 95% ethanol, 50% ethanol, 10% ethanol, and 3% acetic acid (Figure 3a). Both Irganox 1310 and 2,4-DTBP were found in all four food simulants, with migration levels increasing proportionally to the ethanol concentration. Migration amounts ranged from 1.87 × 10^−3^ to 68.13 × 10^−3^ mg/kg for Irganox 1310 and from 25.4 × 10^−3^ to 370.8 × 10^−3^ mg/kg for 2,4-DTBP. Irganox 1076, Irgafos 168, and Irganox 1010 were exclusively detected in 95% ethanol. The variation in AOs migration can be attributed to their octanol/water partition coefficients; AOs with higher coefficients migrate more readily in lipid-like media, and the high ethanol concentration in 95% ethanol markedly enhances their migration [41]. Furthermore, AOs tend to migrate more into lipid media than aqueous media, consistent with previous studies [4,6,42]. As shown in Figure 3b,c, increasing migration temperature and duration results in higher AOs migration amounts, with temperature exerting a more pronounced influence than time. Moreover, due to distinct solubility and volatility, different AOs are differentially influenced by temperature during migration, corroborating prior research [43,44,45]. The impacts of microwave exposure time and power levels, as well as ultraviolet irradiation time, on AO migration indicate that the migration of different AOs exhibits less variability under these treatments compared to untreated conditions (Figure 3d–f), thereby corroborating previous findings [46]. Compared to microwave and UV exposure, factors such as the type of food simulant, temperature, and time exert a more significant influence on the migration of AOs in PLA-based and starch-based products. This suggests that the material structure remains relatively stable under microwave and short-term UV exposure. Consequently, greater consideration should be given to the type of food, contact time, and temperature when using such tableware. For instance, it is advisable to avoid using these products with high-fat foods, at high temperatures, or for extended periods. It should be noted that this investigation was conducted using a limited number of representative samples; therefore, the findings related to microwave heating and UV exposure may not be generalizable to all varieties of biodegradable tableware. Furthermore, the testing employed only a 6 cm^2^ sample piece, which may not accurately represent the actual contact geometry of complex items such as bowls, knives, and forks.

### 3.3. Migration Results of AOs from Products into Soybean

Based on the migration results obtained in 95% ethanol, eight representative samples exhibiting high DR and AO migration levels were selected for further analysis. These samples included two PLA-based samples (one plate and one box) and six starch-based samples (one box, one bowl, one fork, and three additional boxes), all of which are hot-selling and represent typical product types. Among these samples, six AOs were identified: Irganox 1310, Antioxidant JX-35, Irganox 1076, Irgafos 168, Irganox 1010, and 2,4-DTBP. Their DRs ranged from 25% to 100%, while their migration amounts varied between 4.81 × 10^−3^ and 4.23 mg/kg. Pretreatment of these six AOs in soybean oil was performed using a simple and cost-effective freezing degreasing method. Subsequently, an exposure risk assessment of the AOs in the eight representative samples was conducted.

A series of parameters were systematically optimized, including solvent type, oil sample mass, ultrasonic extraction time, and freezing time. MeOH and ACN are commonly utilized as extraction solvents for AOs in soybean oil [9]. Given the favorable solubility of Irgafos 168 in DCM, this study investigated the impact of six solvents on the peak areas of AOs: ACN, ACN:DCM (9:1), ACN:DCM (7:3), MeOH, MeOH:DCM (9:1), and MeOH:DCM (7:3). The results indicated that ACN alone provided optimal extraction efficiency for AOs, with the exception of Irganox 1076 (Figure S1a). This outcome can be attributed to the properties of ACN, a polar solvent with low lipophilicity, which effectively minimizes the extraction of fats and pigments, thereby reducing matrix interference during AOs extraction. The effects of varying oil sample masses (0.25, 0.5, 1, 1.5, and 2 g) and ultrasonic extraction durations (0, 5, 10, 15, and 20 min) on peak areas are presented in Figure S1b,c. The findings reveal that extraction efficiency is maximized using 0.25 g of oil sample and an ultrasonic extraction time of 15 min. Additionally, the freezing step is critical in the pretreatment process, as it effectively removes lipophilic substances such as fats, thereby purifying the extract [9]. The influence of freezing durations (0, 15, 30, 45, 60, and 75 min) on peak area demonstrated that extraction efficiency for the six AOs was optimal after 60 min (Figure S1d). Consequently, the optimized pretreatment conditions for detecting six AOs in soybean oil were established as follows: ACN as the extraction solvent, 0.25 g oil sample, 15 min ultrasonic extraction, and 60 min freezing time.

The findings showed that the |ME| of six AOs in soybean oil exceeded 50%, indicating significant matrix suppression effects. In the analysis of complex matrix samples, the matrix effect significantly impacts the accuracy of analytical results. Therefore, a matrix-matched calibration curve was employed to correct for the matrix effect and improve analytical accuracy. Table 3 summarizes the standard curves, linear ranges, R^2^, LODs, LOQs, and precision for the six AOs in soybean oil. The results showed that all six AOs exhibited strong linearity in soybean oil (R^2^ > 0.991). The LODs ranged from 0.01 × 10^−3^ to 3.77 × 10^−3^ mg/kg, while the LOQs ranged from 0.05 × 10^−3^ to 12.57 × 10^−3^ mg/kg. Average recoveries for the six AOs varied from 77.6% to 110.3%, with RSDs between 1.7% and 14.7%. It is important to note that, despite using matrix-matched calibration, the significant matrix inhibition effects observed for all six antioxidants may still result in certain uncertainty in quantitative results. This limitation is anticipated to be addressed through improved sample pretreatment methods and the application of isotope-labeled internal standards for quantification.

The migration of four AOs from eight representative samples into soybean oil was identified, specifically Irganox 1310, antioxidant JX-35, Irganox 1076, and Irganox 1010 (Table 3). Antioxidants JX-35 and Irganox 1010 were detected in seven samples, exhibiting the highest DR of 87.5%, whereas Irganox 1310 and Irganox 1076 were found in one starch-based plate, with detection rates of 12.5%. Among these compounds, Irganox 1010 demonstrated the highest average migration level at 343.1 × 10^−3^ mg/kg, with a maximum migration amount of 603.7 × 10^−3^ mg/kg. The maximum migration amounts for Irganox 1310, Antioxidant JX-35, and Irganox 1076 were 9.4 × 10^−3^ mg/kg, 25.4 × 10^−3^ mg/kg, and 396.5 × 10^−3^ mg/kg, respectively. Compared to simulated migration tests using 95% ethanol, the migration of AOs into soybean oil was lower in the same samples, suggesting that food simulants may overestimate the actual migration levels of AOs. Given that these AOs can migrate from disposable tableware into soybean oil—particularly Irganox 1010, which exhibits a notably high migration amount—there is an urgent need to perform an exposure assessment to evaluate the potential safety risks associated with their migration into fatty foods.

### 3.4. Results of Exposure Assessment

#### 3.4.1. Results of Deterministic Assessments (EU and FDA Methods)

In this study, the maximum dietary exposure concentration, defined as the highest migration amount detected in the migration media, was utilized to calculate the EDI and MOE values for each identified AO. Due to the absence of toxicological data for Irganox 245 and 2,4-DTBP, the TTC method was applied to assess their toxicity. These compounds were classified as Cramer Class I using the Toxtree software applications (version 3.1.0) [31], which corresponds to a TTC threshold value of 0.03 mg/kg bw/day, considered as the NOAEL. The EDI, MOE, and NOAEL for each AO are presented in Table S7. Generally, a calculated MOE value exceeding the target MOE suggests a low and potentially negligible risk.

In the EU method assessment, the MOE values for 10 AOs derived from 39 products in 95% ethanol ranged from 5 to 4,019,310. With the exception of 2,4-DTBP, the MOE values for the remaining nine AOs exceeded the target MOE value of 100, suggesting a low risk to human health. Notably, 2,4-DTBP exhibited the lowest MOE value of 5, owing to its NOAEL being determined via the regulatory-accepted TTC approach. This approach incorporates conservative uncertainty factors—including inter- and intraspecies variability, database limitations, and exposure assumptions—into the exposure threshold to compensate for the absence of compound-specific toxicological data. Consequently, the MOE standard value for this compound is set at 1 [31]. Therefore, despite the low calculated MOE for 2,4-DTBP, it remains above the target MOE, thereby providing an adequate safety margin. Furthermore, the calculated MOE values for Irganox 1310, Antioxidant JX-35, Irganox 1076, and Irganox 1010, which migrated from eight samples into soybean oil, were 19,426, 24,032, 4612, and 32,509, respectively. All these values exceed 100, further indicating a low safety risk to human health.

In the FDA evaluation method, the MOE values for six AOs in representative samples ranged from 904 to 4,243,780, all of which exceeded the threshold value of 100. This finding indicates minimal exposure levels and correspondingly low risk to human health. The application of more precise CF and f_T_ in the FDA method for calculating EDI has resulted in notable differences in EDI and MOE values compared to those derived using the EU method. In exposure assessment employing both EU and FDA deterministic approaches, the assumption that maximum migration equates to daily exposure tends to overestimate actual exposure levels, particularly in the context of disposable tableware with short-term contact. Consequently, the evaluation results should be interpreted as upper-bound estimates rather than accurate reflections of consumer exposure. In conclusion, whether the EU or FDA method is applied, the MOE results consistently demonstrate that all detected exposure levels of AOs pose a low health risk.

#### 3.4.2. Results of Probabilistic Assessment (Monte Carlo Simulation Method)

The RfD of Irgafos 168 is set to 1 mg/kg bw/day as recommended by the EFSA [36]. In the absence of specific RfD for Irganox 1310, Irganox 1076, and Irganox 1010, their TTC thresholds were utilized as the RfD for exposure assessments. Exposure predictions for the EDI and HQ of these AOs were simulated using Oracle Crystal Ball, based on the exposure parameters and models detailed in Table S4. Table 4 provides the average, maximum, and various percentile values of EDI and HQ for AOs in 95% ethanol and soybean oil. The P5 indicates the risk associated with lower exposure levels, whereas the mean, P50, and P75 correspond to moderate exposure levels. Higher exposure risks are represented by the P90, P95, and P99 percentiles [7]. It should be noted that F_abs_ estimated from log K_ow_ may overestimate absorption for high-molecular-weight AOs (e.g., Irganox 1010).

The results of the exposure assessment show that the health risk associated with Irganox 1010 is low under average exposure conditions. However, when 95% ethanol is employed as a food simulant, Irganox 1010 at high concentration levels (P90 and above), indicates EDI > RfD and HQ > 1, suggesting a potential risk to human health. Conversely, for other AOs, EDI < RfD and HQ < 1, indicating a low risk from dietary exposure. In soybean oil, only Irganox 1010 was found at a high concentration (P99 and above), EDI > RfD and HQ > 1, further implying a potential health risk. It is important to note that these conclusions are based on high-percentile conservative exposure scenarios derived from Monte Carlo simulations rather than typical consumer exposure. Nevertheless, enhanced monitoring of Irganox 1010 migration from disposable biodegradable tableware is warranted to safeguard consumer health. Previous research has shown that the EDI of AOs decreases with age, underscoring the necessity to focus on the risks posed to underage consumers who may ingest AOs from such tableware [6,41]. Moreover, degradation products of AOs and the effects of low-dose combined exposure to AOs may also pose health risks [6,47]. Therefore, further in-depth investigations are recommended.

The apparent discrepancy between the low risk indicated by deterministic assessment and the high percentile risk revealed through Monte Carlo simulation primarily stems from the distinct underlying assessment methodologies. The EU/FDA deterministic assessment employs conservative fixed-value parameters and maximum migration calculations to establish a universal safety threshold, thereby yielding a low estimated risk. Conversely, Monte Carlo simulation accounts for extreme scenarios characterized by high migration and exposure levels by iteratively calculating the probability distributions of key parameters; its high percentile risk thus reflects these rare but critical conditions. This divergence does not signify a methodological contradiction but rather highlights the complementarity of the two approaches. Collectively, they not only confirm the safety of routine exposure but also identify potential hazards under extreme conditions, consistent with the findings of the present study. Furthermore, in this study, the Monte Carlo input parameters are primarily based on the accelerated migration tests and worst-case regulatory scenarios. As a result, the high percentile HQ is most sensitive to the migration concentration C and the toxicity threshold (RfD); however, the quantitative contribution of each parameter has yet to be assessed. Future research should employ methods such as Sobol or Morris sensitivity analyses to systematically evaluate the influence of model structure on the study’s conclusions.

## 4. Conclusions

This study evaluated the migration behavior and exposure risk of 20 AOs in 39 disposable biodegradable tableware products using UPLC-MS/MS. The results indicated that the presence of ten AOs was detected in fatty food simulants (95% ethanol). Among these, Irganox 1010 exhibited the highest average migration level (0.29 ± 0.62 mg/kg), followed by Irgafos 168 and Irganox 1076. Notably, nine AOs were identified in starch-based products, with each product containing two or more AOs. The type of food simulant, as well as migration temperature and time, exerted a more pronounced effect on migration than microwave and ultraviolet treatments. Specifically, AOs demonstrated a greater tendency to migrate into high-fat food simulants. Migration of AOs in soybean oil was analyzed using a simple and cost-effective freezing degreasing method, which detected four AOs. Irganox 1010 again showed the highest average migration level at 343.1 × 10^−3^ mg/kg, with a maximum migration of 603.7 × 10^−3^ mg/kg. The migration levels in soybean oil were lower than those observed in 95% ethanol, indicating that 95% ethanol, as a fatty food simulant, tends to overestimate AO migration compared to actual edible oils. Risk evaluations conducted according to EU and FDA methods indicated a low exposure risk associated with the detected AOs. However, Monte Carlo simulation analyses indicated that exposure to high doses of Irganox 1010 in both 95% ethanol and soybean oil could pose health risks. It is important to emphasize that this conclusion pertains to high-percentile conservative exposure scenarios derived from the Monte Carlo simulations. Given the widespread use of disposable biodegradable tableware, enhanced monitoring and regulation of Irganox 1010 migration are imperative. In conclusion, based on the reliable dataset obtained from systematic analysis of 39 products via UPLC-MS/MS and conservative parameter settings in risk assessment, this study provides a scientific framework for the safety evaluation of AOs in disposable biodegradable tableware. Future research should prioritize long-term migration studies, characterization of degradation products, bioavailability assessments, and the coupling migration data with actual consumption surveys to facilitate a comprehensive safety assessment of AOs.

## Figures and Tables

**Table 1:** Information and mass spectrometric parameters of 20 AOs.

Compound	Molecular Mass	Precursor Ion (*m*/*z*)	Product Ion 1, CE (V)	Product Ion 2, CE (V)	Species (Polarity)	RT (min)	Cone Voltage (V)
Irganox 1310	278.19	296.28	167.15, 12	223.18, 8	(M + NH_4_)+	2.93	16
Antioxidant JX-35	292.2	310.3	181.21, 14	237.17, 8	(M + NH_4_)+	3.49	8
Irganox 1222	356.21	357.24	301.25, 14	107.05, 38	(M + H)+	3.36	62
Antioxidant 2246	340.24	339.27	163.16, 28	147.13, 48	(M − H)−	3.76	62
Antioxidant 300	358.2	357.22	194.11, 30	179.12, 48	(M − H)−	3.51	6
Irganox 3052	394.25	412.28	121.06, 22	339.25, 10	(M + NH_4_)+	3.81	12
Antioxidant DLTP	514.41	532.43	143.08, 24	329.32, 16	(M + NH_4_)+	5.57	32
Irganox 1076	530.47	548.56	107.11, 40	149.14, 26	(M + NH_4_)+	6.03	14
Irganox 1024	552.39	570.42	181.26, 42	441.33, 20	(M + NH_4_)+	3.5	44
Irganox 565	588.39	589.35	250.23, 48	289.3, 46	(M + H)+	5.17	98
Irganox 245	586.35	604.38	263.31, 22	177.28, 42	(M + NH_4_)+	3.43	52
Irganox 1098	636.49	637.51	321.39, 40	525.45, 28	(M + H)+	3.55	82
Irgafos 168	646.92	647.48	147.19, 48	235.18, 52	(M + H)+	6.72	20
Irganox 1035	642.4	660.49	249.25, 28	309.27, 22	(M + NH_4_)+	3.86	14
Irganox 697	696.44	714.4	159.18, 52	473.32, 26	(M + NH_4_)+	3.63	2
Irganox 80	740.45	758.29	628.99, 22	163.00, 48	(M + NH_4_)+	3.77	98
Irganox 330	774.6	792.69	219.29, 38	203.34, 80	(M + NH_4_)+	4.83	82
Irganox 3114	783.52	801.48	219.29, 32	203.27, 76	(M + NH_4_)+	4.06	20
Irganox 1010	1176.78	1195.07	219.42, 78	163.28, 78	(M + NH_4_)+	4.38	98
2,4-DTBP	206.71	205.1	189.24, 26	173.02, 4	(M − H)−	3.51	28

CE: Collision energy; RT: Retention time.

**Table 2:** Migration results of 20 AOs from 39 disposable biodegradable tableware products into 95% ethanol (×10^−3^ mg/kg).

AOs	PLA-Based (n = 10)			Starch-Based (n = 15)			Fiber-Based (n = 14)			Total (n = 39)			SML	
	Mean ± SD	Median	Max	Mean ± SD	Median	Max	Mean ± SD	Median	Max	Mean ± SD	Median	Max	PRC ^a^	EU ^b^
Irganox 1010	352.9 ± 881.7	ND	2768.4	530.5 ± 638	203.3	1721.8	ND	ND	ND	294.5 ± 623.7	ND	2768.4	–	–
Irgafos 168	423.4 ± 1338.8	ND	4233.5	390 ± 448.6	235.0	1323.4	ND	ND	ND	258.6 ± 733.0	ND	4233.5	–	10 ^c^
Irganox 1076	46.8 ± 77.1	1.5	227.0	132.2 ± 156.8	46.4	436.6	ND	ND	ND	62.8 ± 117.9	ND	436.6	6	6
2,4-DTBP	37.1 ± 117.3	ND	370.8	27.3 ± 71.9	0.0	228.7	ND	ND	ND	20.0 ± 73.5	ND	370.8	–	–
Antioxidant DLTP	67.4 ± 213.2	ND	674.1	ND	ND	ND	ND	ND	ND	17.3 ± 107.9	ND	674.1	5	5
Irganox 1310	7.3 ± 21.4	ND	68.1	18.5 ± 15.2	16.8	43.5	ND	ND	ND	9.0 ± 16.1	ND	68.1	–	–
Antioxidant JX-35	1.1 ± 3.6	ND	11.4	14 ± 17.2	7.7	59.7	ND	ND	ND	5.7 ± 12.5	ND	59.7	–	–
Irganox 3114	ND	ND	ND	2.9 ± 6.8	0.0	23.8	ND	ND	ND	1.1 ± 4.0	ND	23.8	5	5
Irganox 330	ND	ND	ND	0.3 ± 0.8	0.0	2.8	ND	ND	ND	0.1 ± 0.5	ND	2.8	–	–
Irganox 245	ND	ND	ND	0.04 ± 0.2	0.0	0.6	ND	ND	ND	0.02 ± 0.01	ND	0.6	9	9
Irganox 80	ND	ND	ND	ND	ND	ND	ND	ND	ND	ND	ND	ND	0.05	0.05
Irganox 1222	ND	ND	ND	ND	ND	ND	ND	ND	ND	ND	ND	ND	–	–
Irganox 3052	ND	ND	ND	ND	ND	ND	ND	ND	ND	ND	ND	ND	6	6
Irganox 1024	ND	ND	ND	ND	ND	ND	ND	ND	ND	ND	ND	ND	15	15
Irganox 565	ND	ND	ND	ND	ND	ND	ND	ND	ND	ND	ND	ND	30	30
Irganox 1098	ND	ND	ND	ND	ND	ND	ND	ND	ND	ND	ND	ND	45	45
Irganox 1035	ND	ND	ND	ND	ND	ND	ND	ND	ND	ND	ND	ND	2.4	2.4
Irganox 697	ND	ND	ND	ND	ND	ND	ND	ND	ND	ND	ND	ND	–	–
Antioxidant 300	ND	ND	ND	ND	ND	ND	ND	ND	ND	ND	ND	ND	0.48	0.48
Antioxidant 2246	ND	ND	ND	ND	ND	ND	ND	ND	ND	ND	ND	ND	–	–
∑AOs	93.6 ± 512.1	ND	4233.5	111.6 ± 305	ND	1721.8	ND	ND	ND	66.9 ± 323.9	ND	4233.5		

ND: <LOD; ^a^ GB 9685–2016 for the use of Additives for Food Contact Materials and Products [27]; ^b^ Commission Regulation (EU) No 10/2011 on plastic materials [28]; ^c^ Recommended limits in the latest assessment by the European Food Safety Authority [36].

**Table 3:** Method evaluation for AOs in soybean oil and results of AOs migration from represented samples (n = 6).

Method Evaluation								Migration Amount (×10^−3^ mg/kg)							
AOs	Linear Equation	Range (mg/kg)	R^2^	LOD (×10^−3^ mg/kg)	LOQ (×10^−3^ mg/kg)	Recovery (%)	RSD (%)	No. 1	No. 3	No. 15	No. 16	No. 19	No. 20	No. 21	No. 22
Irganox 1310	y = 134,213.1 x + 3118.8	0.01–0.8	1.000	0.21	0.70	81.0 ^a^	3.9 ^a^	ND	ND	ND	ND	9.4	ND	ND	ND
						87.6 ^b^	2.4 ^b^								
						99.2 ^c^	4.6 ^c^								
Antioxidant JX-35	y = 2,449,746.4 x + 5706.9	0.01–0.8	0.998	0.01	0.05	84.6 ^a^	7.2 ^a^	18.8	ND	13.7	19.5	25.4	6.2	6.1	4.9
						102.5 ^b^	9.5 ^b^								
						103.7 ^c^	7.2 ^c^								
Irganox 1076	y = 414.1 x + 131.9	0.01–0.8	0.992	1.50	5.01	95.0 ^a^	1.7 ^a^	ND	ND	ND	ND	396.5	ND	ND	ND
						80.4 ^b^	8.8 ^b^								
						93.8 ^c^	3.6 ^c^								
Irgafos 168	y = 286.3 x + 94.8	0.01–1	0.992	0.14	0.46	79.2 ^a^	9.0 ^a^	ND	ND	ND	ND	ND	ND	ND	ND
						77.6 ^b^	12.4 ^b^								
						88.0 ^c^	3.2 ^c^								
Irganox 1010	y = 30,798.3 x − 979.7	0.01–1	0.991	0.08	0.27	106.0 ^a^	13.1 ^a^	328.6	ND	221.7	53.9	603.7	379.2	259.2	555.7
						110.3 ^b^	12.2 ^b^								
						107.1 ^c^	3.2 ^c^								
2,4-DTBP	y = 336.2 x + 55.8	0.01–0.8	0.997	3.77	12.57	109.6 ^a^	14.3 ^a^	ND	ND	ND	ND	ND	ND	ND	ND
						85.8 ^b^	14.7 ^b^								
						86.2 ^c^	9.9 ^c^								

ND: <LOD; ^a^ Spiked level: 0.02 mg/kg; ^b^ Spiked level: 0.2 mg/kg; ^c^ Spiked level: 0.5 mg/kg. No. 1. PLA-White meal box; No. 3. PLA-Greyish-white meal box; No. 15. Starch-Off-white meal box; No. 16. Starch-Off-white meal box; No. 19. Starch-Yellow dumpling plate; No. 20. Starch-Off-white small round plate; No. 21. Starch-White small round bowl; No. 22. Starch-Light yellow cutlery.

**Table 4:** EDI and HQ of AOs in different migration mediums using Monte Carlo Method.

	AOs	Mean	P5	P50	P75	P90	P95	P99	RfD
EDI ^a^	Irganox 1310	0.11	0.01	0.05	0.12	0.26	0.39	0.96	9
(×10^−3^ mg/kg bw/d)	Antioxidant JX-35	0.07	0.00	0.03	0.07	0.16	0.26	0.65	9
	Antioxidant DLTP	0.21	0.00	0.04	0.13	0.42	0.85	3.24	30
	Irganox 1076	0.82	0.05	0.39	0.88	1.86	2.88	6.59	9
	Irganox 245	0.00	0.00	0.00	0.00	0.00	0.00	0.00	30
	Irgafos 168	3.11	0.09	1.06	2.95	7.17	12.58	31.65	1000
	Irganox 330	0.00	0.00	0.00	0.00	0.00	0.00	0.01	1.5
	Irganox 3114	0.01	0.00	0.00	0.01	0.03	0.06	0.17	1.5
	**Irganox 1010**	4.00	0.19	1.68	4.05	**9.17**	**15.06**	**37.16**	9
	2,4-DTBP	0.27	0.00	0.07	0.20	0.54	1.04	3.08	30
HQ ^a^	Irganox 1310	12.54	0.80	6.00	13.58	28.69	43.64	107.13	
(×10^−3^)	Antioxidant JX-35	7.82	0.38	3.24	7.99	17.46	29.13	71.75	
	Antioxidant DLTP	7.15	0.05	1.21	4.46	13.94	28.48	108.05	
	Irganox 1076	91.40	5.73	43.55	97.89	206.70	320.48	731.91	
	Irganox 245	0.01	0.00	0.00	0.01	0.02	0.03	0.11	
	Irgafos 168	3.11	0.09	1.06	2.95	7.17	12.58	31.65	
	Irganox 330	0.76	0.01	0.17	0.56	1.60	3.00	9.79	
	Irganox 3114	9.64	0.19	2.56	7.61	21.28	38.39	111.03	
	**Irganox 1010**	444.83	21.51	186.56	450.34	**1019.39**	**1673.77**	**4128.79**	
	2,4-DTBP	8.86	0.15	2.21	6.70	17.97	34.59	102.71	
EDI ^b^	Irganox 1310	0.02	0.000	0.005	0.01	0.03	0.06	0.16	9
(×10^−3^ mg/kg bw/d)	Antioxidant JX-35	0.15	0.03	0.16	0.23	0.30	0.34	0.42	9
	Irganox 1076	0.64	0.02	0.22	0.59	1.44	2.48	6.38	9
	**Irganox 1010**	4.02	0.75	4.28	6.16	7.81	8.81	**10.85**	9
HQ ^b^	Irganox 1310	1.68	0.05	0.55	1.46	3.57	6.21	17.69	
(×10^−3^)	Antioxidant JX-35	16.61	3.01	18.03	25.69	33.25	37.45	46.61	
	Irganox 1076	71.22	2.08	24.20	65.56	160.05	275.36	708.93	
	**Irganox 1010**	446.82	83.17	475.40	684.52	868.04	978.72	**1205.44**	

^a^ EDI and HQ of AOs in 95% ethanol food simulants; ^b^ EDI and HQ of AOs in soybean oil.

## Data Availability

The original contributions presented in this study are included in the article/Supplementary Materials. Further inquiries can be directed to the corresponding author.

## Supplementary Materials

The following supporting information can be downloaded at: https://www.mdpi.com/article/10.3390/foods15050964/s1, Figure S1: Optimization of pretreatment conditions of six AOs in soybean oil. (a) Extraction solvent; (b) Oil sample mass; (c) Ultrasonic time; (d) Freezing time; Table S1: Product information of 39 disposable biodegradable tableware; Table S2: Names, abbreviations, CAS registry numbers, molecular formula and molecular mass of 20 AOs; Table S3: CF and f_T_ of various food contact plastic materials; Table S4: Exposure assessment parameters and models of AOs using Monte Carlo Method; Table S5: Relevant parameters of Monte Carlo simulation exposure assessment; Table S6: Linear parameters, LODs, LOQs, RSDs of 20 AOs in different food simulants; Table S7: Exposure assessment of AOs in typical samples using EU and FDA Methods.
