# Supplementary material for: Migration and Safety Assessment of 20 Antioxidants in 39 Disposable Biodegradable Tableware Products
Source: Foods. 2026 Mar 9;15(5):964. doi: 10.3390/foods15050964 (PMC12984358; doi:10.3390/foods15050964)
Supplement: Supplementary file 1 [file foods-15-00964-s001.zip › foods-4116536-supplementary.pdf]

Supplementary material

Migration and safety assessment of 20 antioxidants in 39 disposable biodegradable tableware

Liqian Wang, Yuting Chen, Xiaomeng Gao, Wenjun Zhou, Guowei Ma, Jingwei Zhang, Di Feng*

Key Laboratory of Geriatric Nutrition and Health (Beijing Technology and Business University), Ministry of Education, Beijing 100048, China; Key Laboratory of Geriatric Nutrition and Health, Beijing Technology and Business University, Ministry of Education, Beijing 100048, China; 17860395785@163.com (L.W.); chenyuting20231005@163.com (Y.C.); spgaoxiong@163.com (X.G.); zhouwenjun020603@163.com (W.Z.); maguoweisxl@163.com (G.M.); meowcree2022@163.com (J.Z.)

Corresponding: fengdi0618@126.com; Tel.: +86-010-68985378

This file contains 7 tables and 1 figure.

Table S1. Product information of 39 disposable biodegradable tableware.

Table S2. Names, abbreviations, CAS registry numbers, molecular formula and molecular mass of 20 AOs.

Table S3. CF and f_T of various food contact plastic materials.

Table S4. Exposure assessment parameters and models of AOs using Monte Carlo Method.

Table S5. Relevant parameters of Monte Carlo simulation exposure assessment.

Table S6. Linear parameters, LODs, LOQs, RSDs of 20 AOs in different food simulants.

Table S7. Exposure assessment of AOs in typical samples using EU and FDA Methods.

Figure S1. Optimization of pretreatment conditions of six AOs in soybean oil. (a) Extraction solvent; (b) Oil sample mass; (c) Ultrasonic time; (d) Freezing time

Table S1. Product information of 39 disposable biodegradable tableware.

PLA-based (n = 10)			Starch-based (n = 15)			Fiber-based (n = 14)		
No.	Product	Composition	No.	Product	Composition	No.	Product	Composition
1	White meal box	PLA	11	Light yellow fish plate	Corn starch	26	Yellow-brown meal box	Wheat straw pulp
2	Semi-transparent meal box	PLA	12	Light yellow stir-fry plate	Corn starch	27	Light brown cup	Bamboo fiber + PE film
3	Greyish-white meal box	PLA	13	Light yellow fork and spoon	Corn starch	28	Yellow-brown cup	Paper pulp + PE film
4	Milky straw	PLA	14	Beige meal box	Starch + PP	29	Yellow-brown cup	Paper pulp + PE film
5	Milky straw	PLA	15	Off-white meal box	Starch + PP	30	Yellow-brown snack bowl	Wood pulp + bamboo pulp
6	Light yellow meal box	PLA	16	Off-white meal box	Starch + PP	31	Yellow-brown plate	Sugarcane/bamboo pulp
7	Light grey fork	PLA	17	White meal box	Starch + PP	32	Yellow-brown cup	Fiber + PE film
8	Transparent film	PLA	18	Bright beige meal box	Starch + PC	33	White cup	Paper + PLA film
9	White tea bag	PLA	19	Yellow dumpling plate	Starch	34	White round plate	Sugarcane pulp
10	Transparent cup	PLA	20	Off-white small round plate	Starch	35	White round plate	Sugarcane + wheat pulp
			21	White small round bowl	Starch	36	White dinner plate	Sugarcane pulp
			22	Light yellow cutlery	Starch	37	White round plate	Sugarcane + bamboo pulp
			23	White soup spoon	Starch	38	White straw	Wood pulp
			24	Green dessert spoon	Corn starch	39	Lemon-floral bowl	Paper pulp
			25	Light yellow shopping bag	Starch + PE			

PLA: Polylactic acid; PP: Polypropylene; PC: Polycarbonate; PE: Polyethylene.

Table S2. Names, abbreviations, CAS registry numbers, molecular formula and molecular mass of 20 AOs.

Analyte name	Abbreviation	CAS No.	Molecular formula	Molecular mass
diethyl 3,5–di–tert–butyl–4–hydroxybenzyl phosphate[a]	Irganox 1222	976–56–7	$C_{19}H_{33}O_4P$	356.21
2,2'–methylenebis(4–methyl–6–tert–butylphenol) [a]	Antioxidant 2246	119–47–1	$C_{23}H_{32}O_2$	340.24
4,4'–thiobis(6–tert–butyl–m–cresol) [a]	Antioxidant 300	96–69–5	$C_{22}H_{30}O_2S$	358.2
2–propanoic acid,2–(1,1–dimethylethyl)–6–[[3–(1,1–dimethylethyl)–2–hydroxy–5–methylphenyl]methyl]–4 [a]	Irganox 3052	61167–58–6	$C_{26}H_{34}O_3$	394.25
dilauryl thiodipropionate [a]	Antioxidant DLTP	123–28–4	$C_{30}H_{58}O_4S$	514.41
octadecyl 3–(3,5–di–tert–butyl–4–hydroxyphenyl)propionate [a]	Irganox 1076	2082–79–3	$C_{35}H_{62}O_3$	530.47
2',3–Bis[[3–[3,5–di–tert–butyl–4–hydroxyphenyl]propionyl]]propionohydrazide [a]	Irganox 1024	32687–78–8	$C_{34}H_{52}N_2O_4$	552.39
2,4–Bis(octylthio)–6–(4–hydroxy–3,5–di–tert–butylanilino)–1,3,5–triazine [a]	Irganox 565	991–84–4	$C_{33}H_{56}N_4OS_2$	588.39
triethylene glycol bis(3–tert–butyl–4–hydroxy–5–methylphenyl)propionate [a]	Irganox 245	36443–68–2	$C_{34}H_{50}O_8$	586.35
3,3'–bis(3,5–di–tert–butyl–4–hydroxyphenyl)–N,N'–hexamethylenedipropionamide [a]	Irganox 1098	23128–74–7	$C_{40}H_{64}N_2O_4$	636.49
tris(2,4–di–tert–butylphenyl)phosphite [a]	Irgafos 168	31570–04–4	$C_{42}H_{63}O_3P$	646.92
3,5–bis(1,1–dimethylethyl)–4–hydroxybenzenepropanoic acid thiodi–2,1–ethanediyl ester [a]	Irganox 1035	41484–35–9	$C_{38}H_{58}O_6S$	642.4
(1,2–dioxoethylene)bis(iminoethylene)bis(3–(3,5–di–tert–butyl–4 hydroxyphenyl)propionate) [a]	Irganox 697	70331–94–1	$C_{40}H_{60}N_2O_8$	696.44
3,9–bis[1,1–dimethyl–2–[(3–tert–butyl–4–hydroxy–5–methylphenyl)propionoxy]ethyl]–2,4,8,10–tetraoxaspiro[5.5]decane [a]	Irganox 80	90498–90–1	$C_{43}H_{64}O_{10}$	740.45
1,3,5–trimethyl–2,4,6–tri(3,5–di–tert–butyl–4–hydroxybenzyl)benzene [a]	Irganox 330	1709–70–2	$C_{54}H_{78}O_3$	774.6
tris(3,5–di–tert–buty1–4–hydroxybenzyl) isocyanurate [a]	Irganox 3114	27676–62–6	$C_{48}H_{69}N_3O_6$	783.52
pentaerythritol tetrakis(3–(3,5–di–tert–buty1–4–hydroxyphenyl) propionate) [a]	Irganox 1010	6683–19–8	$C_{73}H_{108}O_{12}$	1176.78
2,4–di–tert–butylphenol	2,4-DTBP	96–76–4	$C_{14}H_{22}O$	206.71
3–(3,5–di–tert–butyl–4–hydroxyphenyl)propionic acid	Irganox 1310	20170–32–5	$C_{17}H_{26}O_3$	278.19
methyl 3–(3,5–di–tert–butyl–4–hydroxyphenyl)propionate	Antioxidant JX-35	6386–38–5	$C_{18}H_{28}O_3$	292.2

[a] Antioxidant is permitted by GB 9685-2016 [1].

2,4-DTBP and Irganox 1310: Degradation products of antioxidant 168 and Irganox 1010; JX-35: Parent compound of Irganox 1076 and Irganox 1010.

Table S3. CF and f_T of various food contact plastic materials.

Category	CF	f_T			
		Aqueous	Acetic	Alcohol	Fatty
PLA	0.05	0.01	0.97	0.01	0.01
Starch-based	0.4	0.49	0.16	0.01	0.34
Fiber-based	0.4	0.49	0.16	0.01	0.34

PLA: Polylactic acid; CF: Consumption factor; f_T: Food type distribution factor.

Table S4. Exposure assessment parameters of AOs using Monte Carlo Method.

Parameter	Unit	Value	Compliance probability distribution
C	mg/kg	Table S5	Table S5
fc	kg/d	1.06	Constant
EF	d/year	6 d/w*4 w/m*12 m/y = 288	Constant
ED	year	76	Constant
LT	d	365 d/y*76 y = 27740	constant
BW	kg	60.95	constant
EDI	mg/kg bw/d	$\mathrm{EDI} = \frac{\mathrm{C} \times fc \times F_{abs} \times EF \times ED}{\mathrm{LT} \times \mathrm{BW}}$	-
RfD	mg/kg bw/d	Table S5	constant
HQ	-	$\mathrm{HQ} = \frac{\mathrm{EDI}}{\mathrm{RfD}}$	-

C: Dietary concentration; fc: Food consumption; EF: Exposure frequency; ED: Exposure duration; LT: Lifetime; BW: Body weight; EDI: Estimated daily intake; RfD: Reference dose; HQ: Hazard quotient; F_{abs}: Absorption factor.

Table S5. Relevant parameters of Monte Carlo simulation exposure assessment.

Food simulants	Antioxidant	Mean±SD ($\times 10^{-3}$ mg/kg)	Probability distribution	K_{ow}	F_{abs}	Cramer Class	RfD (mg/kg bw/day)
95% Ethanol	Irganox 1310	8.98±16.10	Lognormal distribution	4.6	0.91	II	0.009
	Antioxidant JX-35	5.66±12.51	Lognormal distribution	5	0.92	II	0.009
	Antioxidant DLTP	17.28±107.94	Lognormal distribution	9.6	0.95	I	0.030
	Irganox 1076	62.83±117.86	Lognormal distribution	10.2	0.95	II	0.009
	Irganox 245	0.02±0.01	Lognormal distribution	6.1	0.93	I	0.030
	Irgafos 168	258.57±732.96	Lognormal distribution	4.8	0.91	-	1.000
	Irganox 330	0.10±0.49	Lognormal distribution	17.2	0.97	III	0.0015
	Irganox 3114	1.13±4.04	Lognormal distribution	8.9	0.95	III	0.0015
	Irganox 1010	294.54±623.66	Lognormal distribution	19.6	0.97	II	0.009
	2,4-DTBP	20.00±73.52	Lognormal distribution	5.2	0.92	I	0.030
Soybean Oil	Irganox 1310	1.18±3.33	Lognormal distribution	4.6	0.91	II	0.009
	Antioxidant JX-35	11.81±8.84	Normal distribution	5	0.92	II	0.009
	Irganox 1076	49.56±140.18	Lognormal distribution	10.2	0.95	II	0.009
	Irganox 1010	300.25±214.83	Normal distribution	19.6	0.97	II	0.009

SD: Standard deviation; K_{ow}: Octanol/water partition coefficient; F_{abs}: Absorption factor; RfD: Reference dose.

Table S6. Linear parameters, LODs, LOQs, RSDs of 20 AOs in different food simulants.

Food simulant	Antioxidant	Linear equation	Linear range (μg/kg)	R^2	LOD (× 10^{-2} μg/kg)	LOQ (× 10^{-2} μg/kg)	RSD (%)
3% acetic	Irganox 1310	y = 4514x + 27175.3	0.5-50	0.997	0.4	1.5	3.2
	Antioxidant JX-35	y = 53050.1x – 25427.4	0.5-100	1.000	0.7	2.3	4.6
	Irganox 1222	y = 74442.4x – 15679.9	0.5-100	1.000	1.8	6.1	2.5
	Antioxidant 2246	y = 498.6 x - 19255.2	40-2000	1.000	157.5	525.0	3.6
	Antioxidant 300	y = 130.8x - 491.2	0.5-100	0.997	30.3	100.9	5.7
	Irganox 3052	y = 7634.7 x - 1613789.2	40-10000	0.996	376.7	1255.5	4.6
	Antioxidant DLTP	y = 2652.5x + 48511.8	0.5-100	0.997	0.2	0.8	4.2
	Irganox 1076	y = 4789.4x + 20925.2	10-400	0.986	8.0	26.6	4.3
	Irganox 1024	y = 3261.4x - 11301	2-100	0.998	12.7	42.3	5.6
	Irganox 565	y = 433.9x + 796.3	2-100	0.998	8.8	29.4	4.5
	Irganox 245	y = 6984x – 28828.7	0.5-100	0.993	3.5	11.6	3.5
	Irganox 1098	y = 3374.9x - 734805	10-10000	0.995	24.6	81.9	5.4
	Irgafos 168	y = 285.4x + 18331	10-2000	0.990	7.8	26.0	4.7
	Irganox 1035	y = 8743.9x - 12961	0.5-100	1.000	9.0	29.8	5.7
	Irganox 697	y = 3335x - 9309.5	0.5-100	0.998	5.2	17.2	5.3
	Irganox 80	y = 1678.8x - 3098.2	2-100	1.000	14.5	48.5	6.4
	Irganox 330	y = 2233.5x + 5438	0.5-100	0.995	5.3	17.6	4.9
	Irganox 3114	y = 7384 x - 1092611.6	10-10000	0.998	9.4	31.3	7.9
	Irganox 1010	y = 368.2x + 914.7	10-400	0.990	12.9	43.1	2.6
	2,4-DTBP	y = 3.9287x + 205.2	0.5-500	1.000	15.3	51.2	4.5

LOD: Limits of detection; LOQ: Limits of quantitation; RSD: Relative standard deviation.

Continuation sheet

Food simulant	Antioxidant	Linear equation	Range (μg/kg)	R^2	LOD ($\times 10^{-2}$ μg/kg)	LOQ ($\times 10^{-2}$ μg/kg)	RSD (%)
10% ethanol	Irganox 1310	y = 4573x + 42035.6	0.5-500	0.996	0.8	2.5	3.4
	Antioxidant JX-35	y = 53876.3x + 67051.1	0.5-100	0.999	0.7	2.3	6.3
	Irganox 1222	y = 81520.1x + 86725.4	0.5-100	0.999	1.6	5.3	2.9
	Antioxidant 2246	y = 1386.9x - 695465	40-10000	0.973	49.3	164.5	3.6
	Antioxidant 300	y = 2473.6x - 40701	10-500	1.000	79.0	263.5	8.3
	Irganox 3052	y = 2134.8 x - 4785	10-200	0.999	376.7	1255.5	4.8
	Antioxidant DLTP	y = 3700.1x - 3791.4	0.5-20	0.998	0.3	1.1	5.0
	Irganox 1076	y = 752.3x +334462	40-2000	0.999	0.9	2.9	4.8
	Irganox 1024	y = 1590.6x - 739.5	0.5-100	0.981	16.3	54.4	4.4
	Irganox 565	y = 1312.8x - 23807	2-500	0.990	5.5	18.3	7.5
	Irganox 245	y = 13942.6x – 103536.7	2-100	0.990	3.0	9.8	4.2
	Irganox 1098	y = 523.4x - 14865	10-200	0.990	44.3	147.5	2.8
	Irgafos 168	y = 23.5x + 2048.4	10-200	0.999	9.4	31.2	3.7
	Irganox 1035	y = 12314.2x - 13019	0.5-20	0.994	1.1	3.6	2.9
	Irganox 697	y = 1334.8x - 267.5	0.5-100	0.997	4.8	16.2	4.8
	Irganox 80	y = 8582.9x - 211191	2-500	0.975	19.5	65.1	5.1
	Irganox 330	y = 479.2x + 787	0.5-10	0.969	2.0	6.8	4.0
	Irganox 3114	y = 3290.6x - 100935	10-200	0.991	1.0	3.2	2.5
	Irganox 1010	y = 19.1x + 6093.3	10-200	0.998	12.3	41.1	7.0
	2,4-DTBP	y = 3.1x + 271.43	0.5-500	0.997	7.6	25.5	8.4

Continuation sheet

Food simulant	Antioxidant	Linear equation	Range (μg/kg)	R^2	LOD (× 10^{-2} μg/kg)	LOQ (× 10^{-2} μg/kg)	RSD (%)
50% ethanol	Irganox 1310	y = 4622.5x + 54310	0.5-500	0.995	0.5	1.8	2.7
	Antioxidant JX-35	y = 73516.7x + 163252.7	0.5-100	0.998	0.3	1.1	3.8
	Irganox 1222	y = 87585.9x + 397419.9	0.5-100	0.993	0.2	0.6	2.4
	Antioxidant 2246	y = 8692.5x + 144807	10-400	0.992	5.3	17.8	4.5
	Antioxidant 300	y = 2395.5x + 25864	0.5-500	0.994	0.4	1.5	3.2
	Irganox 3052	y = 113296.4 x + 954139.1	10-200	0.999	6.9	23.1	1.2
	Antioxidant DLTP	y = 96682.3x + 138293.7	0.5-20	1.000	0.1	0.2	4.5
	Irganox 1076	y = 54924.9 x + 244821.3	10-200	1.000	1.0	3.2	2.5
	Irganox 1024	y = 57331x + 181115	0.5-100	0.990	0.1	0.4	2.4
	Irganox 565	y = 7745.6x + 430.3	0.5-20	1.000	0.4	1.2	3.4
	Irganox 245	y = 103919.2x + 143469.6	0.5-100	0.999	0.2	0.8	3.2
	Irganox 1098	y = 30830.1 x + 864108.9	10-400	0.990	0.5	1.7	2.2
	Irgafos 168	y = 3730x + 97890.4	10-400	0.991	2.8	9.4	3.8
	Irganox 1035	y = 108110.9x + 443587	0.5-100	0.993	0.1	0.3	3.0
	Irganox 697	y = 61296.2x + 225491.5	0.5-100	0.995	0.1	0.2	2.5
	Irganox 80	y = 20619.6x + 96974.6	0.5-100	0.992	0.1	0.5	3.7
	Irganox 330	y = 29391.2x + 147829.8	0.5-100	0.985	0.1	0.4	3.6
	Irganox 3114	y = 104914.2 x + 1066705	10-200	0.997	0.1	0.4	2.2
	Irganox 1010	y = 3732.5x + 3765.5	10-200	1.000	1.6	5.4	2.3
	2,4-DTBP	y = 6.0287x + 386.8	0.5-100	0.978	17.2	57.3	8.3

Continuation sheet

Food simulant	Antioxidant	Linear equation	Range (μg/kg)	R^2	LOD (× 10^{-2} μg/kg)	LOQ (× 10^{-2} μg/kg)	RSD (%)
95% ethanol	Irganox 1310	y = 8633.9x + 14103	0.5-125	0.996	0.3	1.1	4.8
	Antioxidant JX-35	y = 99391.7 x + 270733.4	0.5-125	0.991	0.2	0.6	6.4
	Irganox 1222	y = 136502.6 x + 83324.0	0.5-125	0.997	0.1	0.4	2.3
	Antioxidant 2246	y = 23543.5 x + 89164.4	0.5-125	0.995	0.1	0.2	3.8
	Antioxidant 300	y = 7711.9 x + 10837.9	0.5-125	0.999	0.3	1.0	4.5
	Irganox 3052	y = 184163.6 x + 644487.0	0.5-125	0.993	0.2	0.5	2.3
	Antioxidant DLTP	y = 71475.0 x + 19370383.6	125-750	0.993	0.8	2.8	2.3
	Irganox 1076	y = 86658.7 x + 4553589.8	25-500	0.991	0.5	1.6	4.4
	Irganox 1024	y = 107598.4 x + 282068.6	0.5-125	0.995	0.1	0.3	2.6
	Irganox 565	y = 14815.5 x + 66721.1	0.5-125	0.995	0.04	0.14	6.6
	Irganox 245	y = 141423.1 x + 245821.7	0.5-125	0.998	0.1	0.3	2.0
	Irganox 1098	y = 68584.4 x + 146856.6	0.5-125	0.994	0.1	0.4	2.5
	Irgafos 168	y = 8552.2 x + 3375757.1	125-2000	0.990	2.2	7.4	2.5
	Irganox 1035	y = 174016.6 x + 561402.7	0.5-125	0.992	0.1	0.2	3.1
	Irganox 697	y = 122387.4 x + 357945.9	0.5-125	0.995	0.1	0.3	2.8
	Irganox 80	y = 50082.5 x + 134392.4	0.5-125	0.995	0.2	0.7	2.2
	Irganox 330	y = 119016.6 x + 635730.2	0.5-125	0.991	0.1	0.2	4.8
	Irganox 3114	y = 211077.9 x + 520678.3	0.5-125	0.993	0.01	0.02	2.9
	Irganox 1010	y = 6477.9 x + 3104362.5	125-2000	0.991	19.8	66.0	3.4
	2,4-DTBP	y = 16.0 x + 1085.5	5-1000	0.991	25.9	86.3	9.2

Table S7. Exposure assessment of AOs in typical samples using EU and FDA Methods.

Matrices	Antioxidant	Dietary concentration ($\times 10^{-3}$ mg/kg)	EDI ($\times 10^{-3}$ mg/kg bw /day)		MOE		NOAEL (mg/kg bw/day)
			EU	FDA	EU	FDA	
Food simulants	Irganox 1310	68.13	1.12	0.007	2684	460556	3
	Antioxidant JX-35	59.67	0.98	0.002	10215	4243780	10
	Irganox 1076	436.63	7.16	0.049	4188	615851	30
	Irgafos 168	4233.53	69.46	0.063	835	920844	58
	Irganox 1010	2768.36	45.42	0.167	7089	1926753	322
	2,4-DTBP	370.82	6.08	0.033	5	904	0.03
	Irganox 245	0.62	0.01	–	2933	–	0.03
	Irganox 330	2.81	0.05	–	4019310	–	185
	Irganox 3114	23.79	0.39	–	19981	–	7.8
	Antioxidant DLTP	674.06	11.06	–	31648	–	350
Soybean Oil	Irganox 1310	9.41	0.15	–	19426	–	3
	Antioxidant JX-35	25.36	0.42	–	24032	–	10
	Irganox 1076	396.50	6.51	–	4612	–	30
	Irganox 1010	603.71	9.90	–	32509	–	322

[a] Calculated at the maximum migration amount. EDI: Estimated daily intake; MOE: Margin of Exposure; NOAEL: No Observed Adverse Effect Level; EU: European Union; FDA: Food and Drug Administration.

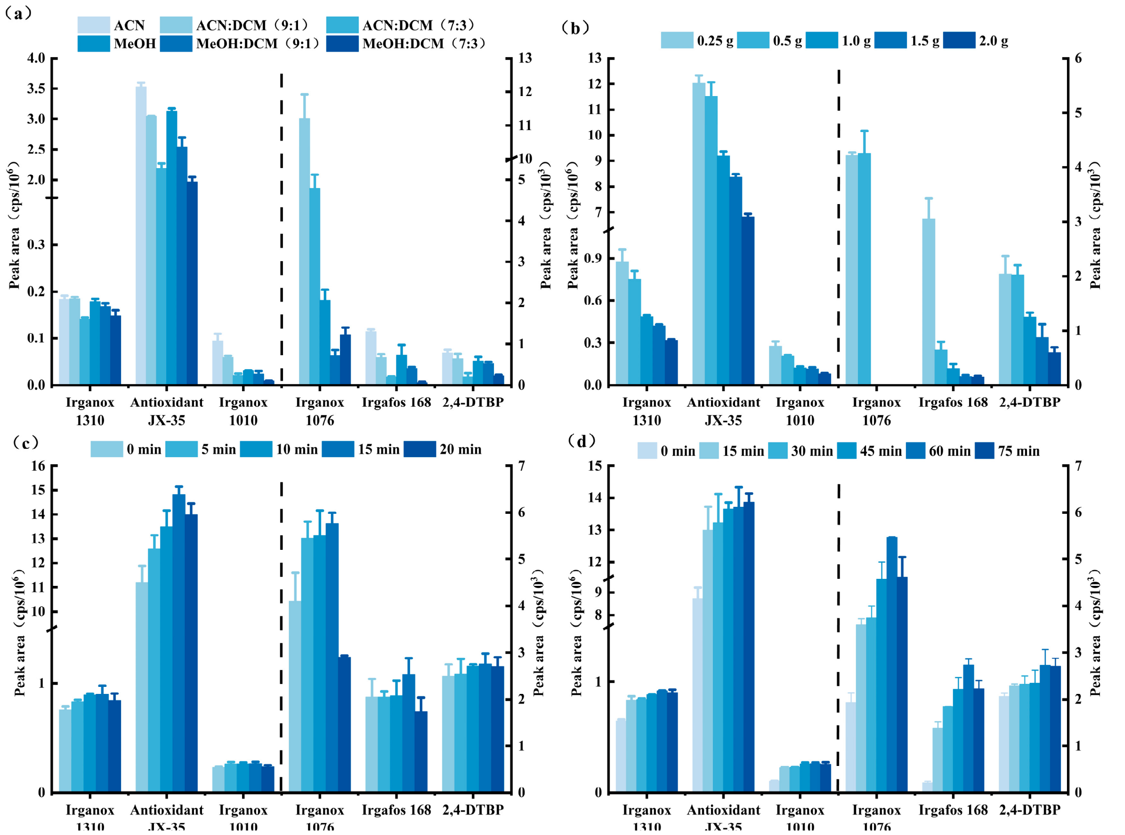

Figure S1. Optimization of pretreatment conditions of six AOs in soybean oil. (a) extraction solvent, (b) oil sample mass, (c) ultrasonic time, (d) freezing duration.

References

1. National Health Commission of the People's Republic of China. (2016). Standard for use of additives for food contact materials and products. Available online: http://9685.foodmate.net/ (Accessed 17 January 2025).
